# Can machine learning improve on the early prediction of upper limb recovery after stroke?

**DOI:** 10.1186/s12984-025-01743-4

**Published:** 2025-10-27

**Authors:** G. J. van der Gun, C. G. M. Meskers, E. R. Andrinopoulou, E. Grauwmeijer, L. Hoogendam, E. E. H. van Wegen, ICAI Stroke Lab, Daniël Bos, Sandra Cornelissen, Ellen Hu, Peter van Hulst, Xi Li, Hester Lingsma, Frank te Nijenhuis, Bob Roozenbeek, Danny Ruijters, Milou Silkens, Ruisheng Su, Sandra Sülz, Theo van Walsum, G. Kwakkel, R. W. Selles

**Affiliations:** 1 Department of Rehabilitation Medicine, Erasmus MC, University Medical Center Rotterdam, Rotterdam, Netherlands; 2 Department of Rehabilitation Medicine, Amsterdam UMC, Vrije Universiteit Amsterdam, Amsterdam, Netherlands; 3 Department of Biostatistics, Erasmus MC, University Medical Center Rotterdam, Rotterdam, Netherlands; 4 Department of Neurorehabilitation, Rijndam Rehabilitation Centre, Rotterdam, Netherlands; 5 Department of Plastic, Reconstructive, and Hand Surgery, Erasmus MC, University Medical Center Rotterdam, Rotterdam, Netherlands; 6 Vrije Universiteit Amsterdam, Amsterdam Movement Science, Rehabilitation and Development, Amsterdam, Netherlands; 7 Vrije Universiteit Amsterdam, Amsterdam Neuroscience, Neurovascular Disorders, Amsterdam, Netherlands

**Keywords:** Stroke, Prognosis, Artificial intelligence, Upper limb, Recovery

## Abstract

**Background:**

Early prediction of upper limb recovery is important to optimise rehabilitation and inform patients but remains challenging due to inter-individual variability. This study aims to (1) develop and validate a machine learning model to predict arm-hand capacity at six months post-stroke using clinical variables from the first week; (2) compare its performance to a mixed-effects model; and (3) co-design a user-friendly output visualisation with clinician input.

**Methods:**

From data of 451 first-ever ischemic stroke patients, we selected total Action Research Arm Test score (ARAT), shoulder abduction, and finger extension as predictors. An XGBoost model was trained on these variables measured at varying time points within the first five months, using 5-fold, 5-repeat cross-validation. We employed bootstrap aggregation to obtain generalisable predictions and prediction intervals to quantify uncertainty. The model's performance was validated on a hold-out set and compared against a mixed-effects model using median absolute error (MedAE).

**Results:**

The XGBoost model achieved a MedAE of 4.2 points (IQR = [1.2, 12.6]) on the ARAT when applied at seven days post-stroke, compared to 13.7 points (IQR = [4.6, 27.8]) for the mixed-effects model in the same patients.

**Conclusion:**

Our model provides significantly more accurate predictions of upper limb recovery, with a 69% error reduction compared to the mixed-effects model. Its ease of use, interpretability, and use of routinely collected clinical data make it suitable for digital clinical workflows. Future research could validate the model in larger, more recent cohorts and explore integrating neuroimaging and temporal features.

**Supplementary Information:**

The online version contains supplementary material available at 10.1186/s12984-025-01743-4.

## Introduction

Accurate, patient-specific predictions of upper limb recovery in the initial weeks after stroke are important [1–3]. They assist in setting realistic treatment goals, planning discharge, improving rehabilitation efficiency, and informing patients [4, 5]. Additionally, such predictions can enhance clinical trial design [6] and, when validated, guide treatment decisions effectively.

Because of a large inter-individual variability, predicting upper limb recovery after stroke is challenging for experienced clinicians [7], leading to the development of numerous prediction models (for reviews, see [8, 9]). Many models follow the “SAFE” paradigm, using shoulder abduction (SA) and finger extension (FE) as key predictors [3, 10–12], often alongside clinical measures such as the Fugl-Meyer upper extremity (FM-UE) for muscle synergies [2, 13–15] or the Action Research Arm Test (ARAT) for motor capacity [1]. Some models also incorporate biomarkers from more complex techniques, such as transcranial magnetic stimulation or neuroimaging, to assess corticospinal tract integrity [11, 15].

Many current models rely on linear or logistic regression analysis, which fails to capture the interindividual variation and non-linear time course of upper limb recovery [8]. These methods assume measurements to be independent or measured at predefined times with non-standard equipment, assumptions that often do not align with real-world clinical settings, resulting in less accurate predictions and limited applicability [16].

Recently, a mixed-effects modelling approach has been proposed as a promising alternative to address these statistical limitations [1]. Currently used in clinical rehabilitation practice [17], this model relies on routinely collected, easy-to-measure clinical scores: SA, FE, and ARAT. It accommodates measurements at varying time points, updating predictions as new data become available throughout recovery. A key strength is its ability to forecast non-linear, patient-specific recovery profiles. However, its accuracy remains limited in the early post-stroke stages, when predictions are most relevant [18].

Several strategies can improve the prediction performance of models applied during the initial weeks post-stroke. One approach is to include additional features, such as patient characteristics, disease severity, or treatment details. Another is to adopt alternative modelling techniques. Contemporary approaches, though not yet widely developed or validated at scale, have shown promise in predicting outcomes in stroke and other domains [8, 19, 20]. These algorithms are particularly effective in handling nonlinear, patient-specific relationships and interactions between input variables and outcomes [21].

An example of such an algorithm is Extreme Gradient Boosting (XGBoost), a machine-learning technique based on the gradient-boosting algorithm [22]. Unlike parametric models, XGBoost does not rely on predefined functional forms, allowing it to flexibly capture complex non-linear relationships and interactions between predictors. XGBoost has consistently ranked among the top performers in Kaggle competitions, a widely recognised benchmark for machine learning performance [23]. Recently, XGBoost has been shown to outperform other models in classifying outcomes of global disability following the modified Rankin Scale (mRS), particularly in the acute phase post-stroke [24].

In this study, we develop and internally validate an XGBoost model to predict arm-hand capacity, as measured by the ARAT, at 6 months post-stroke. We hypothesise that this model will demonstrate superior predictive accuracy compared to an existing innovative mixed-effects model when applied in the initial weeks post-stroke on the same patients. Additionally, we aim to co-design a user-friendly and intuitive output visualisation by incorporating feedback from clinicians into the design process.

## Methods

### Study population

As also reported by Selles et al. [1], we combined data from first-ever ischemic stroke patients collected between 2000 and 2019 across 44 Dutch centres in four cohort studies: EPOS [12], EXPLICIT[6], 4D-EEG [25] and EXPLORE. These datasets included ARAT scores, recommended clinical assessments of the most affected upper limb—specifically shoulder abduction (SA) from the Motricity Index and finger extension (FE) from the Fugl-Meyer Upper Extremity motor score—and the corresponding time of measurement in days post-stroke. The 4D-EEG and EXPLORE cohorts had measurements during the first week and then at 5, 12, and 26 weeks. The EXPLICIT study included measurements at 1, 2, 3, 5, 12, and 26 weeks, while the EPOS study recorded measurements within the first 3 days, at days 5 and 9, and 26 weeks. All studies enrolled adult patients with monoparesis or hemiparesis within 72 h of stroke onset, excluding those with significant medical history or severe impairments affecting measurement accuracy.

Patients received standard rehabilitation treatment following Dutch guidelines [26], consistent with international standards. In the EXPLICIT study, patients with an unfavourable prognosis were assigned to electromyography-triggered neuromuscular stimulation, while those with a favourable prognosis received modified constraint-induced movement therapy. As the EXPLICIT trial found no intervention effect on ARAT scores at 6 months, the type of intervention was excluded from the analysis.

**Fig. 1:**
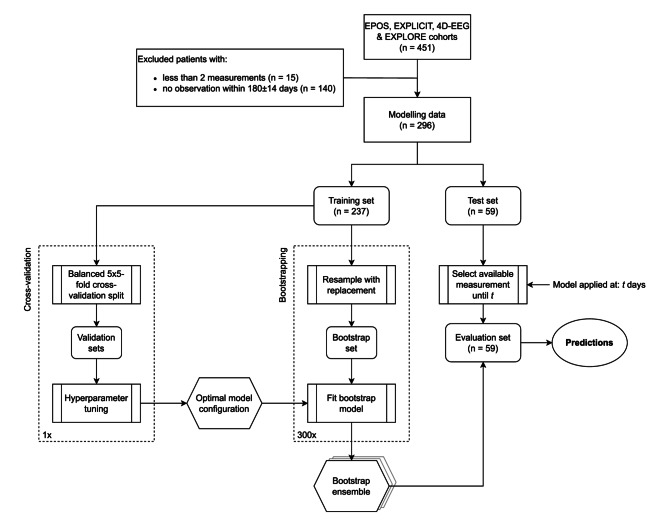
Flow chart depicting the data inclusion and the model-building process. Bootstrapping was employed to generate an ensemble of models that were evaluated on a selection of new, unseen patients from a test set (n=59). All models in the ensemble shared the same configuration, optimised through a 5-fold, 5-repeat cross-validation procedure.

We included patients with at least two serial measurements, with the final measurement taken around 180 ± 14 days post-stroke (see Fig. 1). This approach maximised patient inclusion while ensuring the ability to cross-validate our model.

### XGBoost model development

We developed an XGBoost model to predict patient-specific upper-extremity capacity at 6 months, using ARAT scores as the outcome measure. To compare with Selles et al.'s mixed-effects model [1], we included serial ARAT, SA and FE measurements up to 5 months as predictors. Categorical variables SA and FE were encoded as binary numbers for each level, and the dataset was randomly split into a balanced training (80%) and test set at the patient level.To reduce output variance and improve generalisability, we applied bootstrap aggregation (bagging) to fit an ensemble of XGBoost models on the training set [27]. First, using grid search, we systematically tested different model hyperparameters to find the best configuration, based on 5-fold, 5-repeat cross-validation performance. Then, we generated 300 bootstrap samples from the training set, training one model per sample with the optimal hyperparameters. The final bagged prediction was the median of the predictions from the 300 bootstrap models. Figure 1 illustrates the model-building process.

We used prediction intervals to estimate uncertainty in individual XGBoost predictions. Unlike confidence intervals, prediction intervals account for both model misspecification and uncertainty in predicting individual outcomes. They represent the range within which new observations from the same patient are likely to occur, which is critical for clinicians [28]. Since deriving prediction intervals analytically for non-parametric models like XGBoost is not feasible, we applied methods from Hastie et al. [29], which estimates prediction uncertainty based on the variance in the prediction errors within each bootstrap sample.

### Model comparison

To ensure a fair comparison, we retrained the mixed-effects model by Selles et al. [1] on our training set, maintaining the original structure with total ARAT score, SA, FE, and their measurement times as predictors. For both models, we calculated the absolute error between predicted and observed ARAT scores in the test set. Error distributions were visualised with box plots, showing the median, interquartile range (IQR), and whiskers (Q1—1.5 * IQR, Q3 + 1.5 * IQR), with outliers identified outside this range. The models' error distributions at one week post-stroke were compared using a Wilcoxon Signed-Ranks test. Additionally, we assessed their performance at 1, 6, and 13 weeks post-stroke for patient subgroups with lower, medium, and higher baseline ARAT scores.

All analyses were conducted using RStudio 2024.04.1 + 748 with R version 4.3.1. The "xgboost" and "caret" packages were used for fitting the XGBoost model, and "nlme" for the mixed-effects model.

### Prediction visualisation

In our study, we recognise the importance of presenting model output in an intuitive and user-friendly manner for clinical implementation. To ensure the interface meets clinicians' needs, we recruited clinicians treating stroke patients from two Dutch centres via email to complete an online questionnaire. Participants were required to have clinical experience in stroke care, but practical experience with clinical scores like ARAT, SA, or FE was not required.

The questionnaire was divided into three parts. In the first part, we presented three designs for displaying model outputs, without specifying their individual components (e.g., prediction intervals, current measurements). Participants reviewed these designs, each depicting model outputs for realistic yet fictional patient cases, and responded to four open-ended questions regarding their perceptions of each design. In the second part, we provided explanations for the design, including what they were meant to depict and the meaning of their individual components, after which participants were asked to select their preferred design. Finally, participants viewed an example of additional clarifying annotations and indicated their preference for the interface with or without them. Open feedback on the designs and annotations was also collected. The preferred design was selected by majority vote. The full questionnaire is available in the Supplemental Information.

### Online tool

We implemented the winning interface design using the "shiny" package in R to create an online tool for real-time upper limb recovery predictions. Clinicians and researchers can upload a file with ARAT, SA and FE measurements for a stroke patient to receive automatic predictions of upper limb recovery at 6 months, along with 80% prediction intervals. We selected 80% intervals to balance confidence and practicality, avoiding excessively wide intervals. The source code for our model is publicly available, allowing other centres to develop similar prediction tools tailored to their patient populations.

## Results

### Study population

Of the 451 patients from the four cohort studies (see Table 1), we included 296 who had at least two measurements, with the final measurement taken within ± 14 days of the 6-month mark. Among these patients, 55% had baseline measurements within the first 72 h of stroke onset and 94% within the first two weeks. At their first measurement, 67% of patients had an ARAT score of 10 or lower, indicating a strongly right-skewed distribution. The mean ARAT score at 6 months was 34 (SD = 24). No missing data were observed for the included patients.

**Table 1:** Patient characteristics at baseline (first available measurement: Mean = 5 days, SD = 4.6 days)

		Mean (SD) or n (%) N = 451
Age [years]		65 (14)
Sex [females]		210 (48%)
Type of stroke (Bamford classification)		
	Lacunar Cerebral Infarct (LACI)	215 (49%)
	Partial Anterior Circulation Infarct (PACI)	148 (34%)
	Total Anterior Circulation Infarct (TACI)	73 (17%)
Treatment with recombinant tissue plasminogen activator		
	Yes	104 (23%)
	No	346 (77%)
	Missing	1 (0.2%)
Affected bodyside (right)		177 (40%)
Dominant hand (right)		416 (92%)
National Institutes of Health Stroke Scale (NIHSS) [0–42]		8 (5)
Baseline Action Research Arm Test (ARAT) [0–57]		14 (19)
Baseline Shoulder Abduction		
	No random movement	149 (33%)
	Random activity palpable, no movement visible	44 (10%)
	Random movement seeable but not seeable in total movement range	101 (22%)
	Random movement across total movement range, not possible against resistance	23 (5%)
	Random movement against resistance, but weaker than contralateral side	93 (21%)
	Normal strength in comparison with contralateral side	41 (9%)
Baseline finger extension		
	None	243 (54%)
	Partial	90 (20%)
	Full	118 (26%)
Baseline Fugl-Meyer upper extremity [0–66]		25 (22)
Motricity index arm [0–100]		38 (34)
Motricity index leg [0–100]		49 (32)
NIHSS 8—sensation		
	No sensory loss	203 (45%)
	Mild to moderate sensory loss	187 (42%)
	Severe or total sensory loss	61 (14%)
NIHSS 11—extinction and inattention (neglect)		
	No abnormality	283 (63%)
	Visual, tactile, auditory, spatial or personal inattention	75 (17%)
	Profound hemi-inattention or extinction to more than one modality	93 (21%)

**Fig. 2:**
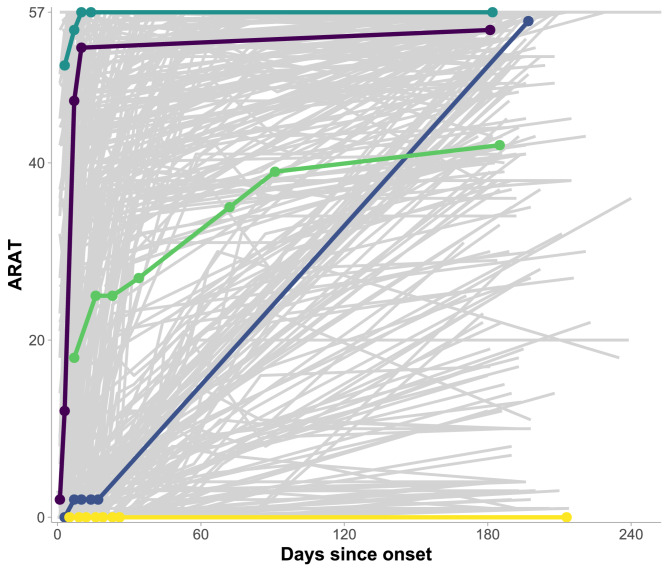
The ARAT recovery profiles of all 451 patients, with individual measurements represented by the dots and five distinct patients highlighted. The longitudinal evolutions of ARAT scores show considerable variation among stroke patients, both in their initial capacity and subsequent measured recovery patterns. ARAT, Action Research Arm Test

Figure 2 displays the ARAT progression over time for all patients and highlights five individuals with distinct baseline ARAT scores and recovery patterns. These cases showcase the diverse and non-linear nature of upper extremity recovery after stroke, with some patients reaching maximum ARAT scores within the first weeks, while others show minimal or no improvement.

### Prediction performance

**Fig. 3:**
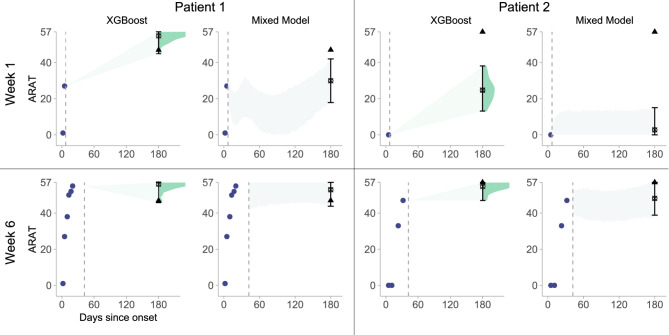
Six-month ARAT predictions and corresponding 80% prediction intervals generated using the XGBoost ensemble and the mixed-effects model for two representative patients (columns) at 1 week and 6 weeks post-stroke (rows). The prediction application time points are indicated with vertical dashed lines in the plot. Purple dots represent available ARAT measurements up to that time point for each patient. Prediction intervals are depicted with error bars and dark-shaded distributions. The light-shaded area visually links the time of prediction to the predicted 6-month ARAT score and does not have statistical meaning. Model predictions are marked by a star (⦻), and the observed (true) outcomes are marked by a triangle (▲). The outputs of the two models differ considerably; the mixed-effects model frequently has true observations falling outside its 80% prediction interval, and the width of its interval remains constant, regardless of the model application time or type of patient. Conversely, XGBoost’s output better matches the observed (true) outcomes, and its prediction interval reflects actual prediction uncertainty, adapting more effectively to different patients and time points. ARAT, Action Research Arm Test

The XGBoost and mixed-effects models, both fitted on a training set of 237 patients (see Fig. 1), produce considerably different outcomes for the same patients in the test set. Figure 3 shows examples of 6-month ARAT predictions and corresponding 80% prediction intervals for both models at two different time points post-stroke for two typical patients. Patient 1, shown in the left column, represents a group of patients with relatively low prediction errors for both models. Patient 2, in the right column, exemplifies a 'difficult' case with higher prediction errors. The XGBoost model provides narrower prediction intervals for Patient 1 compared to Patient 2, and these intervals narrow over time as more recent measurements are included. In contrast, the mixed-effects model shows relatively constant prediction uncertainty regardless of time point or patient type, leading to prediction intervals that frequently exclude the observed (true) outcome. For instance, at 2 weeks post-stroke, 79% of observed outcomes fell within XGBoost’s 80% prediction interval, compared to 66% for the mixed-effects model.

**Fig. 4:**
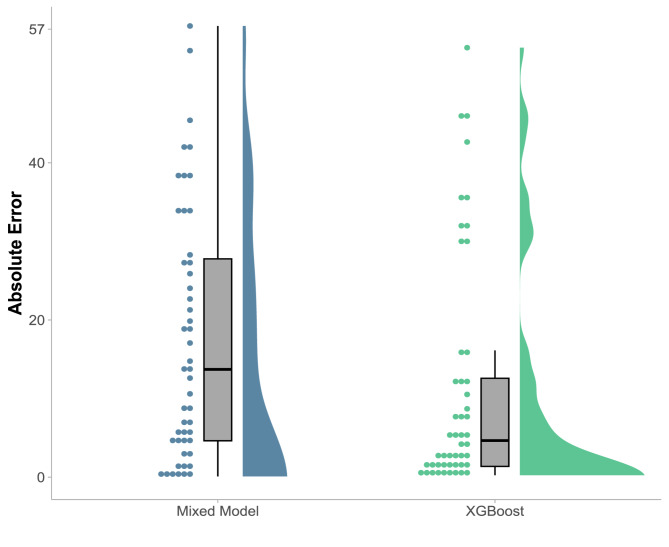
'Rainfall' plots comparing the 6-month prediction errors of the mixed-effects model (blue, left) and the XGBoost model (green, right) applied within the first week post-stroke to 49 patients from the test set. Prediction errors are expressed on the ARAT scale, reflecting the absolute difference between predicted and observed scores. The boxplots display the median, interquartile range (IQR), lower whisker (Q1—1.5 * IQR), and upper whisker (Q3 + 1.5 * IQR). Outliers are defined as values outside the range [Q1—1.5 * IQR, Q3 + 1.5 * IQR]. Individual patient errors are represented by dots, and the error distribution is shown in the lateral density plots. Overall, the XGBoost model demonstrated significantly reduced prediction errors compared to the mixed-effects model

**Fig. 5:**
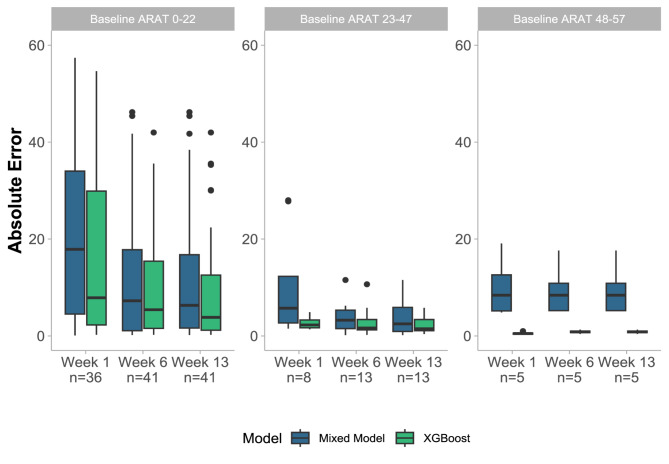
Evolution of 6-month prediction errors of both models when applied at different time points post-stroke on patients from the test set, stratified based on low, moderate, and high baseline ARAT scores (thresholds defined solely for illustrative purposes). Shown are the median, interquartile range (IQR), lower whisker (Q1—1.5 * IQR), and upper whisker (Q3 + 1.5 * IQR). Values outside the range [Q1—1.5 * IQR, Q3 + 1.5 * IQR] were identified as outliers. Prediction errors were highest for patients with low baseline ARAT scores and decreased when the models were applied later post-stroke. The XGBoost performs better than the mixed-effects model in the initial week post-stroke and for patients with medium to low initial motor impairment, as indicated by higher baseline ARAT scores. ARAT, Action Research Arm Test

Figure 4 compares the overall prediction performance of both models in the first week post-stroke using 49 patients from the test set in terms of Median Absolute Error (MedAE). The XGBoost model achieved a MedAE of 4.3 points (IQR = [1.2, 12.6]) for the six-month ARAT score. In comparison, the mixed-effects model had a MedAE of 13.7 points (IQR = [4.6, 27.8]), representing a statistically significant reduction of 69% (p < 0.002) in MedAE for XGBoost. By 6 weeks post-stroke, the MedAE of the XGBoost model further decreased to 2.7 points (IQR = [0.6, 10.9]) (see Fig. 5).

Figure 5 illustrates the 6-month prediction errors of both models across patient subgroups categorised by baseline ARAT scores—low, medium, and high—at various post-stroke time points. Prediction errors were highest for patients with low baseline ARAT scores and decreased as the models were applied later post-stroke. In contrast, the model error remained relatively constant for medium and high baseline ARAT subgroups. Notably, the XGBoost model performs better than the mixed-effects model during the initial week post-stroke, and for patients with high initial motor impairment.

Supplementary Fig. S1 shows the learning curves of the XGBoost model, indicating that performance on both the test and validation sets stabilises before reaching the maximum training sample size (n = 237). This suggests that adding more patients with similar characteristics is unlikely to further improve the model's performance. Supplementary Fig. S2 evaluates model performance based on the number of serial measurements included as predictors. The results demonstrate that incorporating multiple serial measurements, rather than using only the most recent measurement, does not significantly improve predictive performance for either the mixed-effects model or the XGBoost model. Supplementary Figure III presents the SHAP values (Shapley Additive Explanations) of the predictors included in the XGBoost model, showing that baseline ARAT is the main driver for predicting ARAT at 6 months in our model.

### Prediction visualisation

**Fig. 6:**
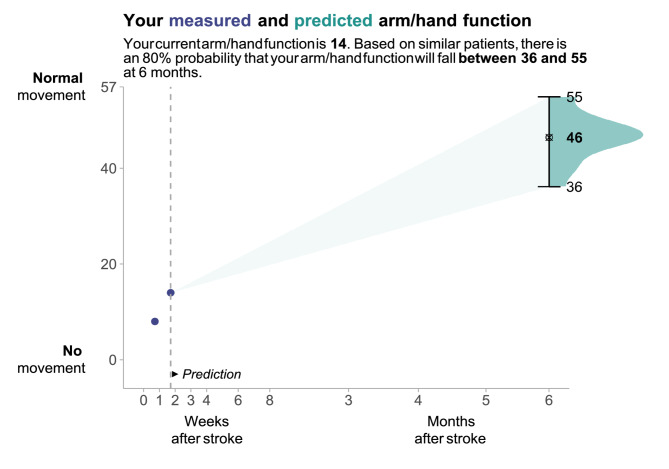
Visualisation of the interface design selected by clinicians through a majority vote from three alternatives. The dashed vertical line indicates the time of model application, here shown at 11 days post-stroke. Purple dots represent available ARAT measurements up to this point. The 80% prediction interval is depicted with an error bar and a dark-shaded lateral distribution, which is the probability density distribution of the potential ARAT outcomes. The upper and lower boundaries of the prediction interval and the final bagged point prediction are labelled. This visualisation was designed to be intuitive and patient-friendly for use in consultations

Of the 59 participants invited, 20 responded to the questionnaire on interface design. Most were occupational therapists (n = 12), followed by four rehabilitation physicians, three physiotherapists, and one psychologist. The design receiving the most votes (40%) is shown in Fig. 6. Respondents favoured this design for its balance of information density and clarity. However, some found the lateral density distribution of potential ARAT outcomes (dark green shading) unclear without explanation. 90% preferred annotations above the graph with labels indicating the most likely ARAT score and prediction interval boundaries. Additionally, 65% deemed the output suitable for patient consultations, though three respondents suggested further explanation would be needed to assist patient understanding.

## Discussion

Aiming to improve on an innovative mixed-effects model [1], we developed an XGBoost model for predicting 6-month upper-limb recovery in stroke patients. With a minimal predictor set—ARAT, shoulder abduction, and finger extension—the XGBoost model achieved a median prediction error of 4.3 ARAT points when applied on day seven post-stroke. This error is below the minimal clinically important difference of 6 ARAT points at 6 months [30] and represents a 69% reduction compared to the mixed-effects model evaluated at the same time point for the same patients (see Fig. 4).

Despite being trained on the same data using the same predictors, the XGBoost and mixed-effects model yield notably different predictions for the same patients. In particular when applied early post-stroke, the mixed-effects model demonstrates higher prediction errors and often generates uncertainty intervals that exclude the true values. In contrast, the XGBoost model’s 80% prediction intervals correctly encompass the expected proportion of observed values, better reflecting prediction uncertainty. Moreover, although the mixed-effects model can estimate recovery trajectories, these estimates often exhibit fluctuating patterns not typically seen in clinical practice.

Numerous models have been proposed for predicting stroke recovery, yet many fall short in accuracy or face other limitations. Classical and logistic regression models struggle to capture non-linear and patient-specific recovery patterns [8, 16], and typically require balanced data, making them less suitable for the irregular measurement timing that is common in clinical practice. Aside from the mixed-effects model, the most notable exception is the PREP2 model, which correctly classifies 75% of ARAT outcomes at 3 months when applied within the first 7 days [11]. However, it requires the time-consuming and non-standard transcranial magnetic stimulation in one-third of patients. Furthermore, it limits granularity by grouping patients into only four outcome categories that rely on somewhat arbitrary thresholds.

The XGBoost model developed in this study overcomes several limitations of existing models and offers notable advantages. Unlike parametric models, XGBoost inherently captures interaction effects, inter-individual variability, and non-linear progression that is typical of upper limb recovery post-stroke [30]. It accommodates measurements taken at non-fixed time points and can be applied at any time post-stroke. Additionally, our model is based on easily measurable variables routinely collected in clinical practice, allowing for straightforward integration into existing workflows, which is essential for implementation success [31]. Finally, incorporating clinician feedback, we designed intuitive prediction visualisations and implemented them in an online tool, facilitating testing by clinicians and researchers of other centres.

While XGBoost does not model within-patient correlations, it performs well in the initial weeks post-stroke, where repeated measurements are often limited. Functional scores such as the ARAT typically show minimal short-term change, and as shown in Supplemental Figure II, multiple early measurements offer little advantage over a single recent measurement. Nevertheless, future research could explore models such as Recurrent Neural Networks that combine temporal modeling with the flexibility of machine learning approaches.

Despite significant improvements in overall prediction performance, our model still shows suboptimal results for a small subset (~ 5%) of patients with baseline ARAT scores below 10 points. Similar to other models [11, 12], our model appears to interpret some voluntary shoulder or finger movement as indicative of a favourable prognosis. When patients recover without these indicators, or conversely, do not recover despite their presence, it results in significant prediction errors. This observation is consistent with prior research [13]. While prediction intervals help flag high-uncertainty cases, it remains crucial to address the prediction errors for this particular patient phenotype. The inability of a powerful machine learning algorithm to detect these specific patterns from a sufficiently large training sample (see Supplemental Fig. S1) suggests that the clinical variables considered in this study do not fully capture the information needed to identify these outliers.

This study was limited to clinical variables alone, suggesting avenues for future research to enhance predictive performance through additional variables, particularly for outlier cases. First, patients in most studies, including ours, received standard care, which is highly heterogeneous. Including therapy type and dose could address some of this variability in future models. Second, evidence suggests that corticospinal tract integrity, indicated by upper limb motor-evoked potentials, as well as neuroimaging biomarkers captured within the first 24 h, may provide additional predictive value beyond clinical modelling [3, 11, 21, 33–35]. Lastly, deficits in sensory and cognitive domains could also influence motor performance [11], suggesting that incorporating variables related to these domains might provide additional prognostic value.

Based on our model's performance on a test set of data held out from model training, we expect it to generalize well to different populations. We employed a combination of bootstrapping and cross-validation to minimize bias. Furthermore, while our dataset is limited to ischemic stroke patients primarily included before the widespread adoption of endovascular thrombectomy [36], it is diverse and representative of the Netherlands. However, since the results come from the same cohort, external validation is crucial before implementation. As thrombectomy may alter recovery distributions, future research could prioritise evaluating our model with more recent datasets that reflect current treatment standards.

A large proportion of clinical prediction models never reach clinical practice [37] and incorporating clinician feedback early in their development is crucial for enhancing their clinical usability and utility [21, 32, 38, 39]. We applied user-centred design principles to create our prediction visualisation, using structured feedback from clinicians and therapists to improve its interpretability and usability. To gather this feedback, we took a pragmatic approach and invited clinicians from two Dutch centres. While a broader investigation into implementation barriers and facilitators would require a dedicated study, our current approach represents an important first step toward successful clinical integration.

In conclusion, our study demonstrates that machine learning algorithms like XGBoost offer significant added value for accurate, patient-specific motor recovery predictions after stroke. Reviews of prediction tools in stroke show that contemporary methods often fail to surpass classical regression [8, 16], and it may thus be tempting to dismiss machine learning as merely 'old wine in a new bottle'. However, our study illustrates the potential of machine learning by achieving markedly different and more accurate predictions compared to a mixed-effects model trained on the same dataset. Additionally, our approach shows promise for predicting other upper limb recovery measures and outcomes in related domains. Once validated and implemented, such models could support accurate prognoses, helping clinicians set realistic treatment goals, inform patients, plan discharge timing, and guide triage decisions. To unlock the full potential of machine learning in stroke recovery prediction, the next step is the creation of large, international databases with standardised data collected throughout the stroke care pathway [40, 41].

## Supplementary Information

Additional file 1.

## Data Availability

Clinical data are available on reasonable request. The code is publicly available at https://gitlab.com/icai-stroke-lab/XGBoost-ARAT and the online prediction tool at https://icaistrokelab.shinyapps.io/XGBoost-ARAT-Predictions/.
