# Supplementary material for: Can machine learning improve on the early prediction of upper limb recovery after stroke?
Source: J Neuroeng Rehabil. 2025 Oct 27;22:223. doi: 10.1186/s12984-025-01743-4 (PMC12557897; doi:10.1186/s12984-025-01743-4)
Supplement: Supplementary file 1 — Additional file 1. [file 12984_2025_1743_MOESM1_ESM.docx]

# Supplementary Material

Supplemental Figure I displays the learning curves for the XGBoost model, plotting the mean absolute error (MAE) against the training sample size for the training, validation, and test sets. Beyond approximately 100 patients, the learning effect ceases to generalise to the test set, as evidenced by the stabilisation of the MAE for test and validation sets. This indicates that adding more patients with similar characteristics does not further enhance the model's performance.


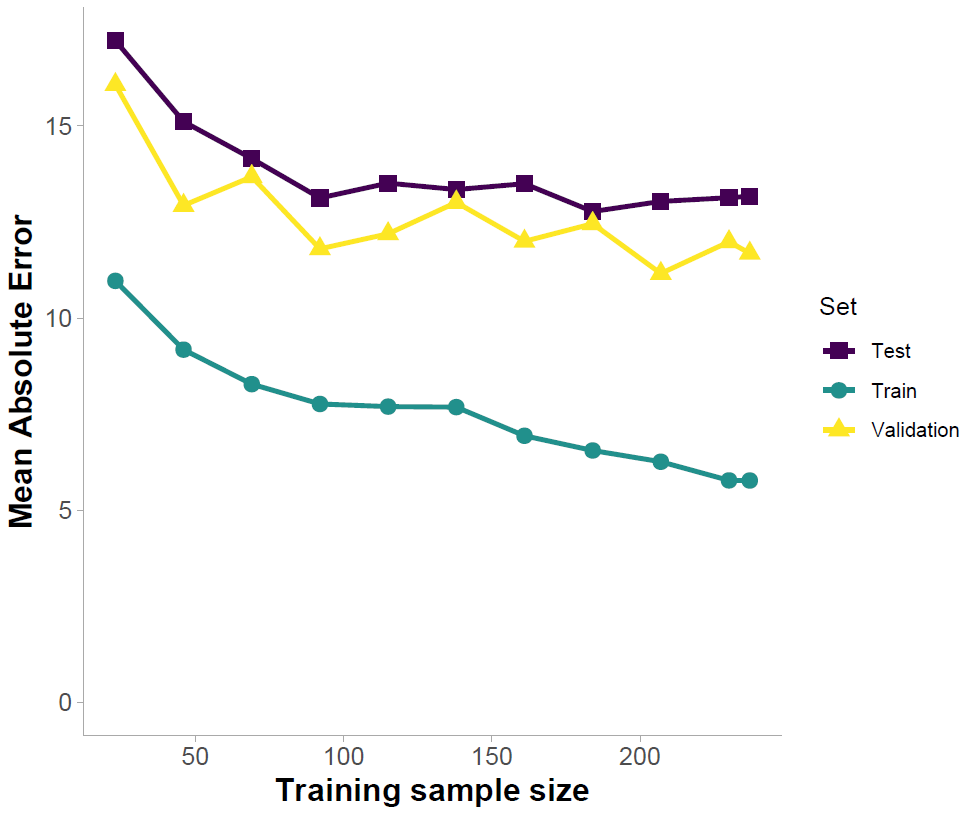


Figure I: Learning curves of the XGBoost model showing mean absolute error (MAE) as a function of training sample size for the training, validation, and test sets. The curves illustrate the performance of the model on the test set (purple ■), train set (green ●), and validation set (yellow ▲). As the training sample size increases, the MAE for the training set initially decreases rapidly, indicating that the model is learning from the data. The MAE for the validation and test sets also decrease, demonstrating improved generalisation performance. At approximately 100 patients, the validation and test errors start to stabilise, suggesting the model's optimal training sample size, beyond which additional data provides diminishing returns in terms of performance improvement.

The cross-validation performance of both models as a function of the number of serial measurements included as predictors is shown in Supplemental Figure II. The error distributions indicate that incorporating multiple serial measurements does not substantially enhance the predictive performance of either the mixed-effects model or the XGBoost model compared to using only the most recent measurement available at the time of model application.


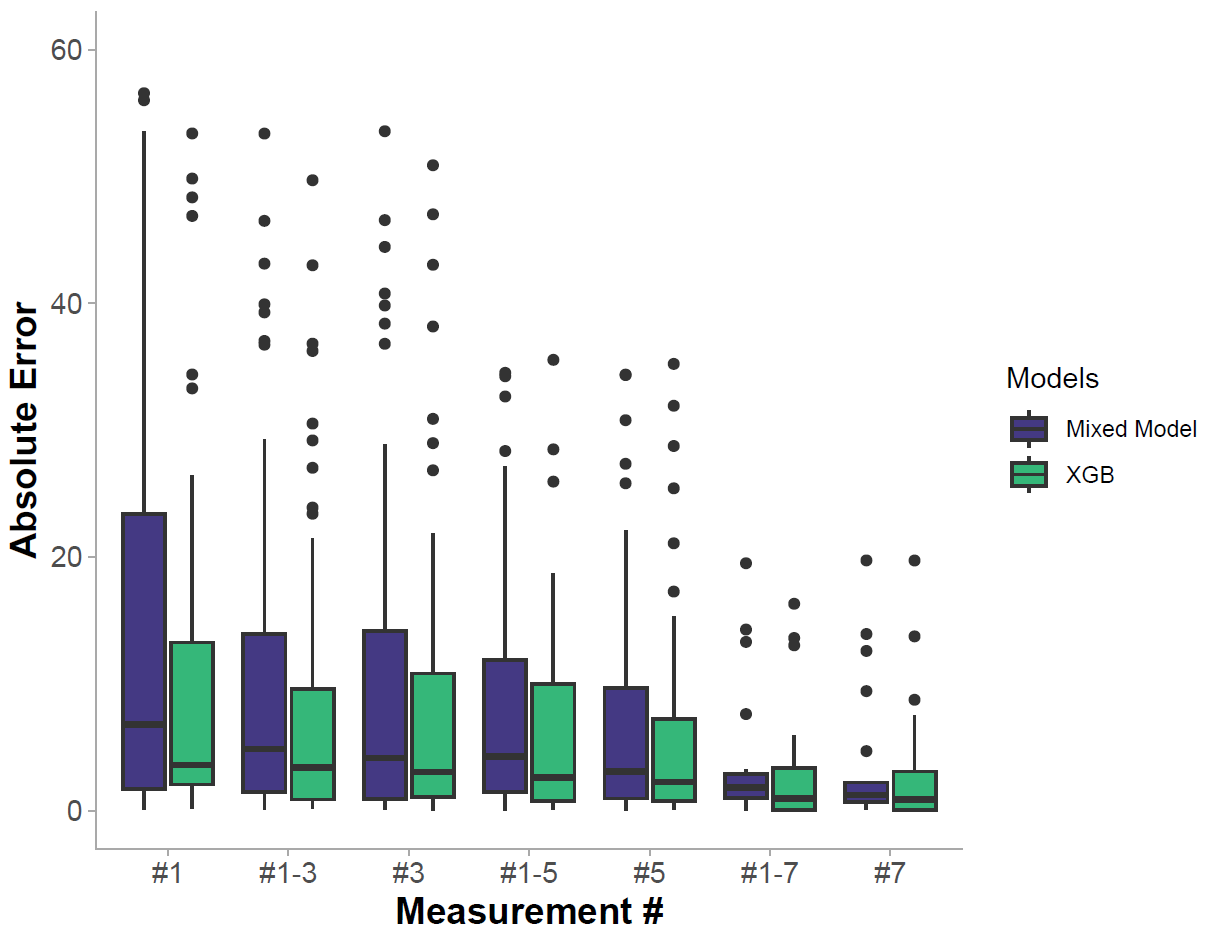


Figure II: Cross-validated model performance, measured in absolute error, as a function of the number of serial measurements included as predictors. For instance, the model's performance using only the third measurement (#3) as a predictor was compared to its performance when the first, second, and third measurements (#1-3) were included as predictors. The results indicate that incorporating multiple serial measurements does not substantially enhance the predictive performance of either the mixed-effects model or the XGBoost model compared to using only the most recent measurement available at the time of model application.

For computational efficiency, only a single best-tuned model was evaluated instead of the bootstrap ensemble. Additionally, for the learning curves, MAE was used over MedAE due to the lack of native support for MedAE in the "caret" package in R. Consequently, the absolute model performance shown in Supplemental Figure I and Figure II is not directly comparable to that of the bagged ensemble, which is reported in the main part of this manuscript.

Supplementary Figure III presents the SHAP values (Shapley Additive Explanations) of the predictors included in the XGBoost model. Baseline ARAT and Days (time from stroke onset to baseline assessment) exhibit the largest average absolute SHAP values, signifying their primary role in predicting ARAT at 6 months. Baseline is defined as a measurement. Baseline is defined as any measurement within the first month.


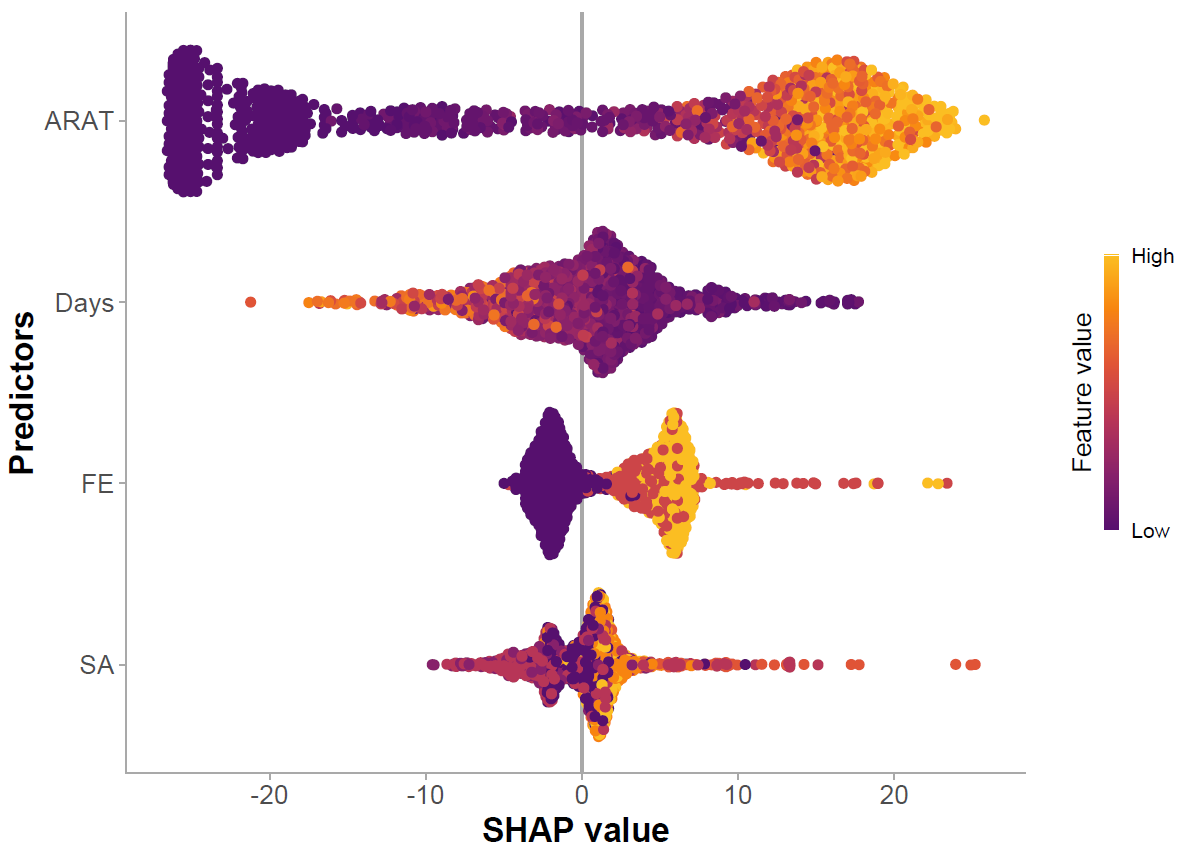


Figure III Feature importance for 6-Month ARAT Prediction. This beeswarm plot visualizes SHAP (Shapley Additive Explanations) values for each predictor in the XGBoost model. Each point represents an individual patient's SHAP value for a given feature, with colour indicating the feature's original value. ARAT, Action Research Arm Test; FE, Fugl-Meyer Finger Extension; SA, Motricity Index Shoulder Abduction; Days, time from stroke onset to assessment.

Supplemental Figure IV provides an overview of the three design prototypes presented to clinicians as part of the questionnaire. Each column represents one design, and each row corresponds to a distinct patient case. Initially, no annotations or explanations were provided for the designs. Clinicians were prompted to answer the following four questions for each design:

- What aspect(s) of this design do you like the most?
- What aspect(s) of this design do you like the least?
- What was the most difficult to understand?
- Would you modify anything? If so, what would you change?

Following this, a detailed explanation of each design element was given. Clinicians were then asked to select their preferred design and provide a rationale for their choice.


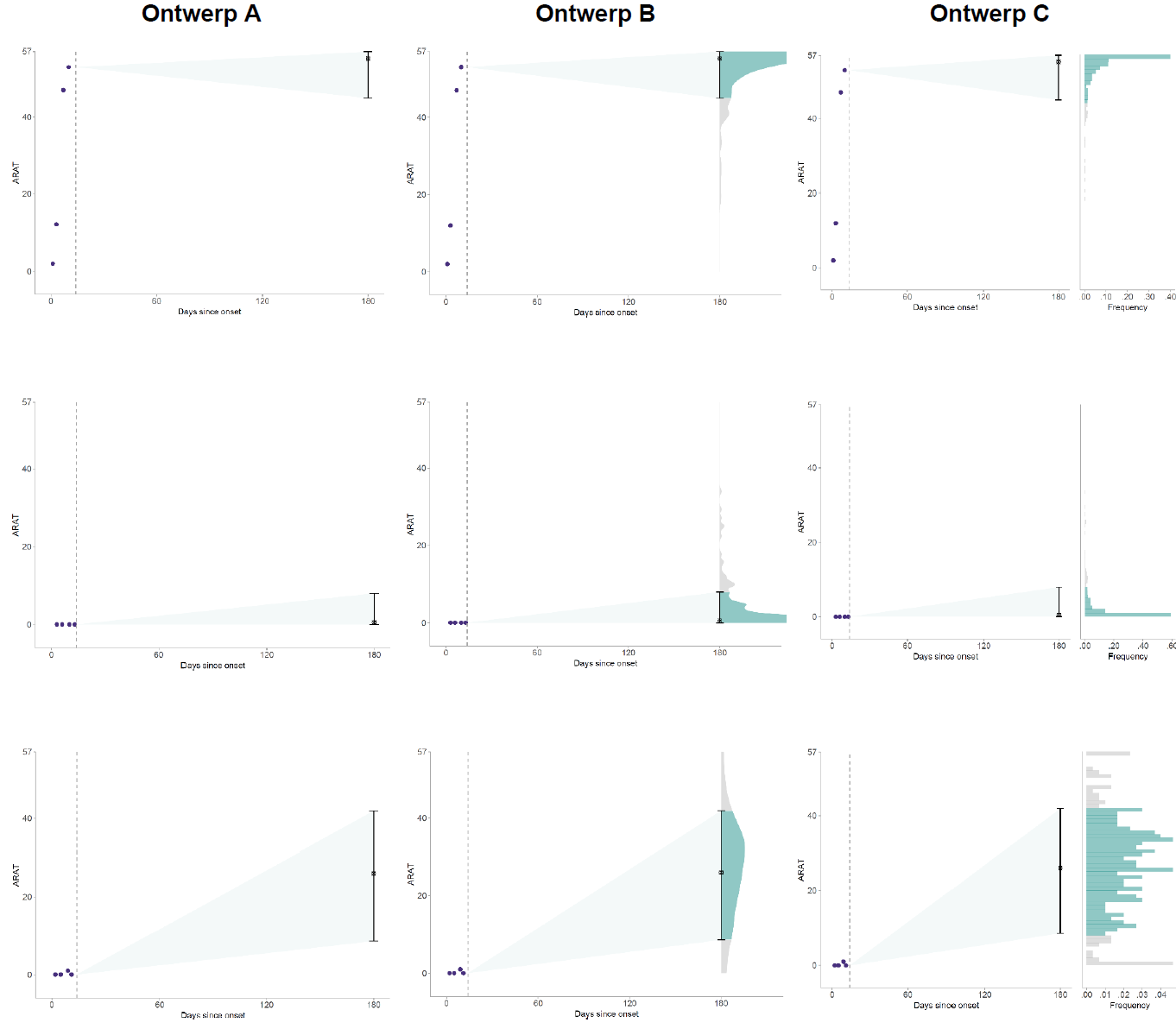


Figure IV An overview of the three designs shown to clinicians in the questionnaire. For each design (columns), three distinct patients were shown (rows). No annotations or additional explanation was provided initially. The clinicians were asked to answer questions about each design before they chose the design that they preferred.
